# Efficacy of *Jasminum subtriplinerve* Extract against 7,12-Dimethylbenz[α]anthracene-Induced Cancer in Mice

**DOI:** 10.4014/jmb.2407.07045

**Published:** 2024-09-20

**Authors:** Phan Hong Minh, Pham Thi Van Anh, Bui Thanh Tung, Ho My Dung, Tran Thi Thu Trang, Pham Thi Hong Nhung, Nguyen Thi Hang, Nguyen Thi Minh Nguyet, Nguyen Viet Phong, Le Ba Vinh, Mai Phuong Thanh

**Affiliations:** 1 University of Medicine and Pharmacy, Vietnam National University, Hanoi, Vietnam; 2 Department of Pharmacology, Hanoi Medical University, Hanoi, Vietnam; 3 National Institute of Medical Materials (NIMM), Hanoi, 11022, Vietnam; 4 Vinmec- VinUni Institute of Immunology, Vinmec Healthcare System, Hanoi, Vietnam; 5 Institute of Marine Biochemistry, Vietnam Academy of Science and Technology, Hanoi, Vietnam; 6 Hanoi University Pharmacy, Hanoi, Vietnam

**Keywords:** Jasminium subtriplinerve Blume, breast cancer, DMBA, IL-2

## Abstract

*Jasminum subtriplinerve* Blume tea is a traditional Vietnamese medicine used to treat impetigo, menstruation issues, and painful menstrual hematometra. Previous studies have shown that extracts and isolated compounds from *J. subtriplinerve* possess diverse pharmacological properties, such as antioxidant, antibacterial, and antidiabetic effects. However, their potential anticancer effects and underlying mechanisms of action have not been clear. Here, we examined the effects of *J. subtriplinerve* extracts against three human cancer cell lines. We also conducted in vivo analyses using a mouse model of 7,12-dimethylbenz[a]anthracene-induced breast cancer, including an investigation of changes in histological sections. The effect of the *J. subtriplinerve* ethyl acetate fraction on cytokine levels (IL-2, PGE2, TNF-α) in serum was determined using ELISA kits. Results showed that the ethyl acetate (EtOAc) fraction had the highest anti-proliferative activity (IC_50_ = 13.7 mg/ml) against the breast cancer (MCF-7) cell line, while the butanol (BuOH) and water fractions did not show any anticancer effects. Additionally, the EtOAc fraction at a dose of 14.4 mg/kg was able to elevate IL-2 levels and suppress the expression of PGE2 in the serum of mice. A remarkable decrease in the percentage of death and tumor incidence in mice was achieved following treatment with the EtOAc fraction at a dose of 14.4mg/kg. No abnormal parameters in blood were observed in the *J. subtriplinerve* treatment groups. These results suggest that *J. subtriplinerve*, when used as tea or a functional food, is nontoxic and has clear chemopreventive effects against breast cancer.

## Introduction

Natural compounds play a crucial role in the development of functional foods and in the early stages of screening for, and the evaluation and development of, new drugs [1]. Such compounds tend to be cost-effective, safe, and efficient, making them highly valuable for use in health supplements and in efforts to identify novel drugs. By leveraging the benefits of natural substances, researchers aim to innovate and improve treatments while ensuring accessibility and benefits for a wide range of individuals.

For example, green tea and coffee contain bioactive compounds that help treat various conditions, including cardiovascular diseases and certain types of cancer [2]. The antioxidants present in these natural products help reduce inflammation and oxidative stress, thereby promoting overall health. Ongoing research into such natural compounds continues to reveal their potential in preventive and therapeutic applications, demonstrating that the integration of natural substances into modern medicine and functional foods can significantly improve public health. This approach enhances treatment efficacy while ensuring safety and affordability.

Cancer remains the primary cause of death worldwide [3]. According to 2019 data from the World Health Organization (WHO), cancer is either the primary or secondary cause of premature death (before the age of 70) in 112 of 183 nations worldwide; it is also ranked third or fourth in an additional 23 countries [4]. Consequently, pharmaceutical companies and independent research institutions around the world prioritize the development of novel cancer drugs. Surgical, chemotherapeutic, radiotherapeutic, targeted, and immunological therapies are currently used to treat cancer [5]. The main goal of chemotherapy is to either completely destroy malignant cells or convert them into benign cells without concurrently damaging healthy cells. Thus, there is an urgent need for the creation of innovative, safe, and effective chemopreventive drugs [6]. Accordingly, the use of medicinal plants as a natural drug source is important.

*Jasminum subtriplinerve* Blume belongs to the jasmine family (Oleaceae) and is widely distributed, particularly in southern Asia [7] (including the southern provinces of China and Hainan Island). Several studies have investigated the chemical composition of *J. subtriplinerve*, which includes the following main components: terpene glycosides, flavonoid glycosides, phenylethanoid glycosides, phenylpropanoid glycosides, steroids, triterpenoids, and phenolics [7-9]. In traditional Vietnamese medicine, *J. subtriplinerve* is believed to have various effects such as clearing heat, relieving rheumatism, activating blood circulation, regulating menstruation, and reducing inflammation. It is used to treat conditions such as amenorrhea, metritis, mastitis, rheumatism, and jaundice. In Vietnam, *J. subtriplinerve* is widely utilized for multiple purposes including improving liver function, increasing bile secretion, stimulating digestion, enhancing appetite, and promoting good sleep. Moreover, it is used to treat rheumatism, bone and joint pain, and skin diseases, as well as snake bites. Its leaves are primarily used to address irregular menstruation, high postpartum fever in women, lymphadenitis, metritis, mastitis, breast abscess, vaginal discharge, rheumatism-induced bone and joint pain, scabies, sores, impetigo, and itchy skin [8-10]. Numerous studies regarding the pharmacological effects of green tea have demonstrated that it has antibacterial, anti-inflammatory, antioxidant, and cell stimulation effects [7]. *J. subtriplinerve*, like green tea, is used daily by local people in Asian countries for similar traditional medicinal purposes. However, their potential anticancer effects and underlying mechanisms of action are unclear.

Here, we explored the effects of extracts of *J. subtriplinerve* leaves on three human cancer cell lines using the MTT assay, as reported in previous studies [11, 12]. We also conducted in vivo analyses using a mouse model of 7,12-dimethylbenz[a]anthracene (DMBA)-induced breast cancer, including an investigation of changes in histological sections.

## Material and Methods

### Materials

Leaves of *J. subtriplinerve* were collected from Nghe An province, Vietnam, in August 2022 and taxonomically identified by Dr. Ngo Duc Phuong. The voucher specimen (accession 2301/TVN-DV) is stored at the Herbarium of the Institute of Traditional Medicine Science in Hanoi, Vietnam.

### Sample Preparation

**Ethanol extract of *Jasminum subtriplinerve***. Dried leaves of *J. subtriplinerve* (7.5 kg) were extracted by reflux with 70% ethanol (three times, 3 h each) at 80°C. The extracts were filtered, combined, and evaporated under reduced pressure, resulting in a green residue (1125.0 g), which was then suspended in water and sequentially partitioned with *n*-hexane, ethyl acetate (EtOAc), and *n*-butanol (BuOH). The fractionated extracts were pooled and evaporated under reduced pressure to yield the respective fractions: an *n*-hexane extract weighing 127.5 g, an EtOAc extract weighing 183 g, an *n*-BuOH extract weighing 155 g, and a water layer obtained after solvent removal.

**Fig. 1:**
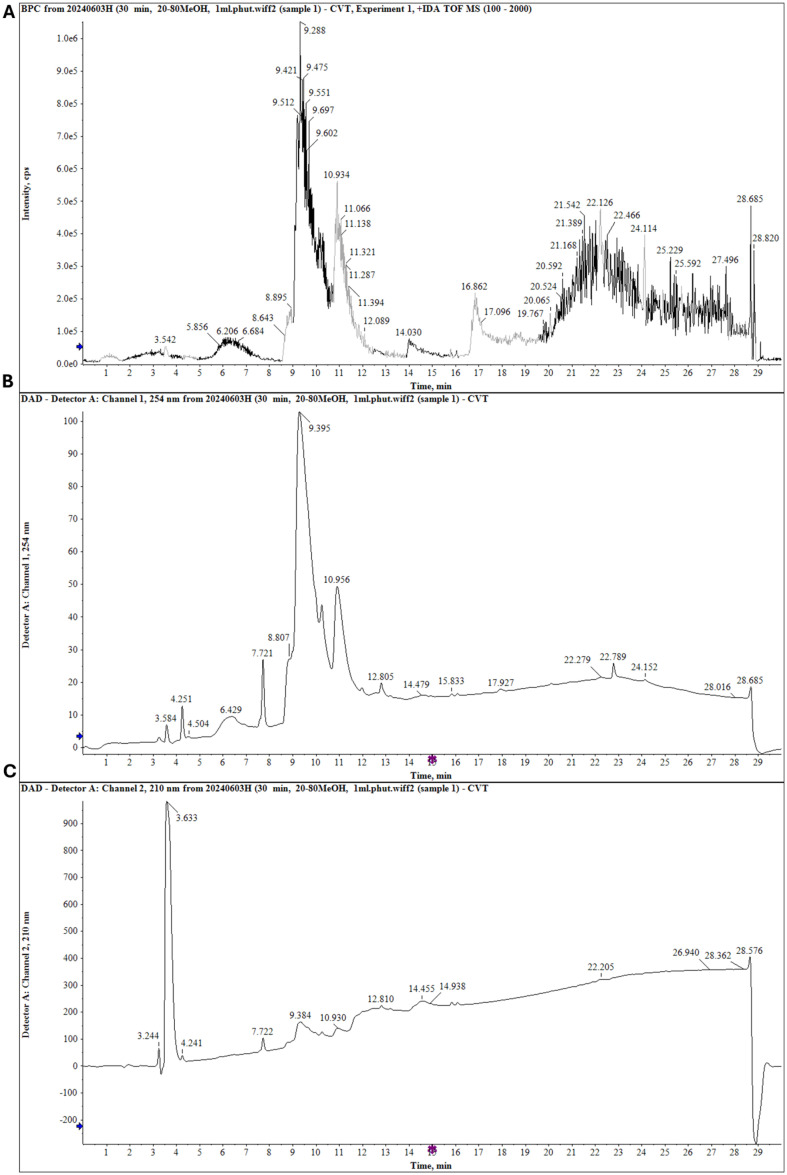
Screening secondary metabolites from an MeOH extract of *J. subtriplinerve* using LC-QTOF MS/MS in positive mode. (**A**) Positive mass (**MS**) chromatogram. (**B**) Ultraviolet (UV; 254 nm) chromatogram. (**C**) Ultraviolet (UV; 210 nm) chromatogram of the MeOH extract from *J. subtriplinerve*.

**Isolation major compounds.** Based on liquid chromatography quadrupole time-of-flight mass spectrometry (LC-QTOF-MS) screening and thin layer chromatography analysis combined with bioassay guidance (Fig. 1), the EtOAc extract was selected for further separation of bioactive compounds. Using combined chromatographic separation techniques, major compounds were isolated from the extract. Briefly, the EtOAc fraction (36.6 g) was subjected to silica gel column chromatography (CC) and eluted with CHCl_3_/MeOH (100:1, 60:1, 40:1, 10:1, and 2:1) to produce six fractions (E1–E6). Compound 1 (500.8 mg) was obtained after fraction E5 had been subjected to silica gel CC and elution with CHCl_3_/MeOH/H_2_O (4:1:0.1, v/v/v), followed by purification on the Sephadex LH-20 CC system using MeOH/H_2_O (4:1, v/v). The structure was identified via NMR analysis, including 1D and 2D NMR, and HR-ESI-MS data.

Acteoside: yellow powder; ^1^H-NMR (600 MHz, CD_3_OD) δ_H_: 4.40 (d, *J* = 8.0 Hz, H-1), 3.41 (dd, *J* = 9.0, 8.0 Hz, H-2), 3.85 (1H, t, *J* = 9.0 Hz, H-3), 4.95 (t, *J* = 9.5 Hz, H-4), 3.55 (m, H-5), 3.56 (1H, m, H-6α), 3.64 (d, J =10.0 Hz, H-6β), 5.20 (d, *J* = 1.5 Hz, H-1'), 3.95 (dd, *J* = 3.1, 1.8 Hz, H-2'), 3.60 (m, H-3'), 3.32 (m, H-4'), 3.60 (m, H-5'), 1.11 (3H, d, *J* = 6.2 Hz, H-6'), 7.08 (d, *J* = 2.0 Hz, H-2''), 6.80 (d, *J* = 8.2 Hz, H-5''), 6.97 (dd, *J* = 8.0, 2.0 Hz, H-6''), 7.62 (d, *J* = 16.0 Hz, H-7''), 6.30 (d, *J* = 16.0 Hz, H-8''), 6.72 (d, *J* = 2.0 Hz, H-2'''), 6.70 (d, *J* = 8.0 Hz, H-5'''), 6.55 (dd, *J* = 8.0, 2.0 Hz, H-6'''), 2.81 (2H, dd, *J* = 13.0, 6.7 Hz, H-7'''), 3.74 (m, H-8α'''), 4.05 (ddd, *J* = 9.5, 8.5, 7.0 Hz, H-8β'''); ^13^C-NMR (150 MHz, CD_3_OD) δ_C_: 104.2 (C-1), 76.0 (C-2), 81.6 (C-3), 70.6 (C-4), 76.2 (C-5), 62.3 (C-6), 103.0 (C-1'), 72.3 (C-2'), 70.4 (C-3'), 73.8 (C-4'), 72.0 (C-5'), 18.4 (C-6'), 127.6 (C-1''), 115.2 (C-2''), 146.8 (C-3''), 149.8 (C-4''), 116.5 (C-5''), 123.2 (C-6''), 148.0 (C-7''), 114.7 (C-8''), 168.3 (C-9''), 131.5 (C-1'''), 117.1 (C-2'''), 146.1 (C-3'''), 144.7 (C-4'''), 116.3 (C-5'''), 121.3 (C-6'''), 36.5 (C-7'''), 72.2 (C-8'''); HR-ESI-MS *m/z* 623.1980 [M-H]^-^ (Calcd. 623.1981).

**Extract preparation for oral gavage.** For in vivo experiments, dried extracts of *J. subtriplinerve* were ground into powder, which was diluted with distilled water to achieve final concentrations of 4.8 g/kg and 14.4 g/kg.

### HPLC Analysis via LC-Q-TOF MS/MS

The major secondary metabolites from *J. subtriplinerve* were screened in accordance with previously reported methods [13, 14]. Briefly, HR-QTOF-MS/MS was performed on an X500 QTOF mass spectrometer (High Performance Benchtop Instruments). The extract was separated using a Capcell Pak C18 analysis column. Solvent A (0.1% v/v formic acid in H_2_O) and solvent B (0.1% v/v formic acid in MeOH) were chosen for the mobile phase. The gradient protocol consisted of increasing MeOH from 20% to 80% over 30 min, with a flow rate of 0.6 ml/min and injection volume of 10.0 μl.

### Cytotoxicity Measurement

The cytotoxic effects of the extract on cancer cell lines were assessed using an MTT assay [15-17]. Briefly, three cancer cell lines, including MCF-7 (human breast cancer), SK-LU-1 (human lung adenocarcinoma), and HepG2 (human hepatoblastoma), were seeded in 96-well plates (Corning, USA) with RPMI medium. After treatment with several amounts of ethanol extract and its fractions (EtOAc, BuOH, and water fractions), the cells were incubated for 48 h at 37°C with 5% CO_2_. Independent experiments were carried out at least three times. Ellipticine was used as a positive control. Data are presented as means ± standard errors (SEs).

The degree of cell inhibition was computed as follows:

$$100\%-\left[\frac{\text{OD}\left(\text{test sample}\right)-\text{OD}\left(\text{blank}\right)}{\text{OD}\left(\text{DMSO control}\right)-\text{OD}\left(\text{blank}\right)}\times 100\right]$$

where OD (blank) is the optical density of the well containing cancer cells without any reagent. IC_50_ values were calculated to evaluate the inhibitory effects of the samples.

### Growth Rate Assay

MCF7 cells were cultured in DMEM supplemented with penicillin G sodium (100 units/ml), streptomycin sulfate (100 μg/ml), amphotericin B (0.25 μg/ml), and 10% fetal bovine serum (FBS). MCF-7 cells were seeded into 12-well plates at a density of 1 × 10^5^ cells per well and then treated with the IC_50_ of the EtOAc fraction or the IC_50_ of ellipticine. Vehicle wells were treated with an equal volume of culture medium. At 2, 6, 24, and 48 h after treatment, live cells were collected and counted using 0.4% trypan blue staining. The number of cells at each time point was recorded.

### Mouse Model of DMBA-Induced Cancer

**Animals.** Female Swiss albino mice aged 7–8 weeks were purchased from the National Institute of Hygiene and Epidemiology, Hanoi, Vietnam, and maintained in the animal center of the Department of Pharmacology, University of Medicine and Pharmacy, Vietnam National University, Hanoi, Vietnam, under pathogen-free conditions. All mice were provided adequate food and water daily. The experimental protocol was approved by the Institutional Animal Ethics Committee of the University of Medicine and Pharmacy, Vietnam National University (Approval number: QG.22.69).

**Experimental design.** Breast tumors were induced using multiple doses of DMBA, which was purchased from Sigma-Aldrich (USA), diluted in olive oil, and administered by gavage. Each animal received 50 mg/kg DMBA weekly for 4 weeks [18].

**Fig. 2:**
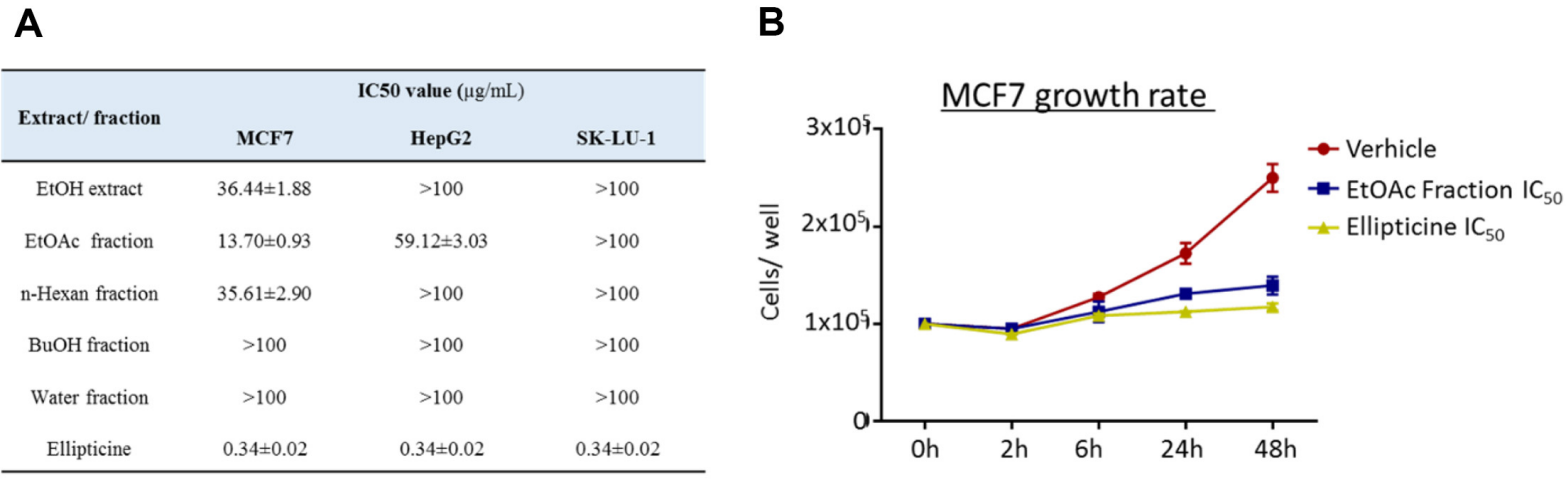
Anti- cancer effects of *J. Subtriplinerve* extract (EtOH) and EtOH fractions in-vitro experiment. EtOH extract and its fractions was treated on three human cancer cell lines in 48 h. The cytotoxicity of *J. Subtriplinerve* on cancer cell lines was assessed using the MTT assay. IC_50_ values was calculated (**A**). Population doubling times are shown (**B**). Data are presented as the means ± SD of three independent experiments.

Briefly, the mice were divided into five groups (*n* = 15 mice each). After acclimatization, the mice were randomly assigned to groups. Group 1 (control group) received only sesame oil vehicle by oral gavage. Groups 2, 3, 4, and 5 were treated with 50 mg/kg DMBA dissolved in sesame oil by oral gavage once weekly for the first 4 weeks of the experimental period; however, group 2 did not receive any additional treatments. During the subsequent 4 weeks, mice in group 3 were treated daily with tamoxifen, whereas mice in groups 4 and 5 were treated with the EtOAc extract of *J. subtriplinerve*. Mice were sacrificed 1 day after the last dose. Blood samples were collected for assays of cytokines and hematological parameters. Tumors in breast and ovarian tissues were collected for morphological analysis (Fig. 2).

**Clinical symptoms.** All mice were monitored daily throughout the experimental period for signs of distress and mortality. Their body weights were measured periodically throughout the experimental period. Hematological parameters were assessed using blood collected from the carotid arteries. Samples (0.3 ml) in ethylenediaminetetraacetic acid (EDTA) were used to measure hemoglobin (Hb) and hematocrit levels; red blood cell (RBC) and white blood cell (WBC) counts; and the percentages of neutrophils, lymphocytes, and platelets. We also estimated liver marker enzymes. For these measurements, blood was collected in non-heparinized tubes and then centrifuged at 3,000 rpm for 10 min. The separated serum was analyzed using the Erba Chem 5v3 Clinical Chemistry Analyzer (Erba Mannheim, India) to measure liver function marker enzymes such as aspartate aminotransferase (AST) and alanine aminotransferase (ALT).

**Histological analysis.** Mice were sacrificed at the end of the eighth week of the experimental period to collect breast and ovarian samples for histological analysis. Additionally, breast and ovarian samples were collected from any mice that died before the specified time point. All tissues were washed with phosphate-buffered saline (PBS), fixed with 10% formalin, processed, and embedded in paraffin blocks. Sections (5 μm) were cut using a microtome (Leica Microsystems, Germany) and stained with hematoxylin and eosin (H&E) for general analysis. Stained sections were observed under an autofocus microscope (Motic, USA) at high power field (HPF) magnification of 200× to confirm cancer status.

**Cytokine measurement.** Blood was collected from each mouse, and serum was isolated by centrifugation. Then, serum samples were prepared for cytokine analyses. Levels of IL-2 and PGE2 were quantified using commercial enzyme-linked immunosorbent assay (ELISA) kits (Bioassay Technology Laboratory, China), in accordance with the manufacturer’s instructions.

**CD3-CD4-CD8 T cell count test.** CD3, CD4, and CD8 T cell populations were detected in mouse draining lymph node cells (dLNs). The dLNs were harvested from mice at the end of the experiment, and single-cell suspensions were prepared. The dLNs (1 × 10^6^ cells) in 100 μl PBS buffer were plated in 24-well culture plates and stained with FITC-labeled anti-CD3, PE-labeled anti-CD4, and PE-labeled anti-CD8 monoclonal antibodies for 20 min at room temperature. These samples were analyzed via flow cytometry.

**Statistical analysis.** Comparisons between the negative control group and treatment groups were performed using nonparametric one-way analysis of variance (ANOVA; *i.e.*, Kruskal-Wallis test) and confirmed with post hoc Bonferroni correction. *P*-values < 0.05 were considered statistically significant, and *p*-values < 0.01 were considered highly significant.

## Results

### Screening of Chemical Constituents via LC-QTOF-MS

The major components of the ethanol extract from the leaves of *J. subtriplinerve* were assessed via liquid chromatography–mass spectrometry (LC-MS). Several major compounds were identified, including phenylethanoid glycosides, flavonoid glycosides, terpen glycosides, triterpenoids, and secoiridoid glycoside, by comparing their retention times, MS/MS fragments, maximum ultraviolet (UV) absorptions (UV max), and molecular weights to those of reference compounds (Fig. 1). Indeed, several secondary metabolites were identified, including astragalin, rutin, isoquercetin, isoquercitrin, kaempferol, rutinoside, nicotiflorin, chevangin A, and jasnervosid A. Next, to identify specific chemicals, we utilized LC-QTOF MS/MS.

### Anticancer Effects in In Vitro Assays

Ethanol extracts and fractions of *J. subtriplinerve* were examined for cytotoxic effects on human cancer cell lines. MCF-7, SK-LU-1, and HepG2 cells were seeded in 96-well plates and treated with various concentrations of these extracts and fractions for 48 h. Cytotoxicity was measured using the MTT assay. Both the full extract and each of its three fractions (EtOAc, *n*-hexane, and *n*-BuOH) significantly inhibited MCF-7 breast cancer cells, with IC_50_ values lower than 50 μg/ml (Fig. 2A). Ellipticine, a well-known anticancer agent, showed IC_50_ values of approximately 1 μg/ml. For MCF-7 cells, the EtOAc fraction exhibited the highest cytotoxicity with an IC_50_ value of 13.70 ± 0.93 μg/ml; other fractions were less effective. In addition, the EtOAc fraction of *J. subtriplinerve* significantly attenuated the proliferation of MCF-7 at IC_50_ in 48 h culture (Fig. 2B).

### Effects of Extract Fractions on Cancer Mortality

**Fig. 3:**
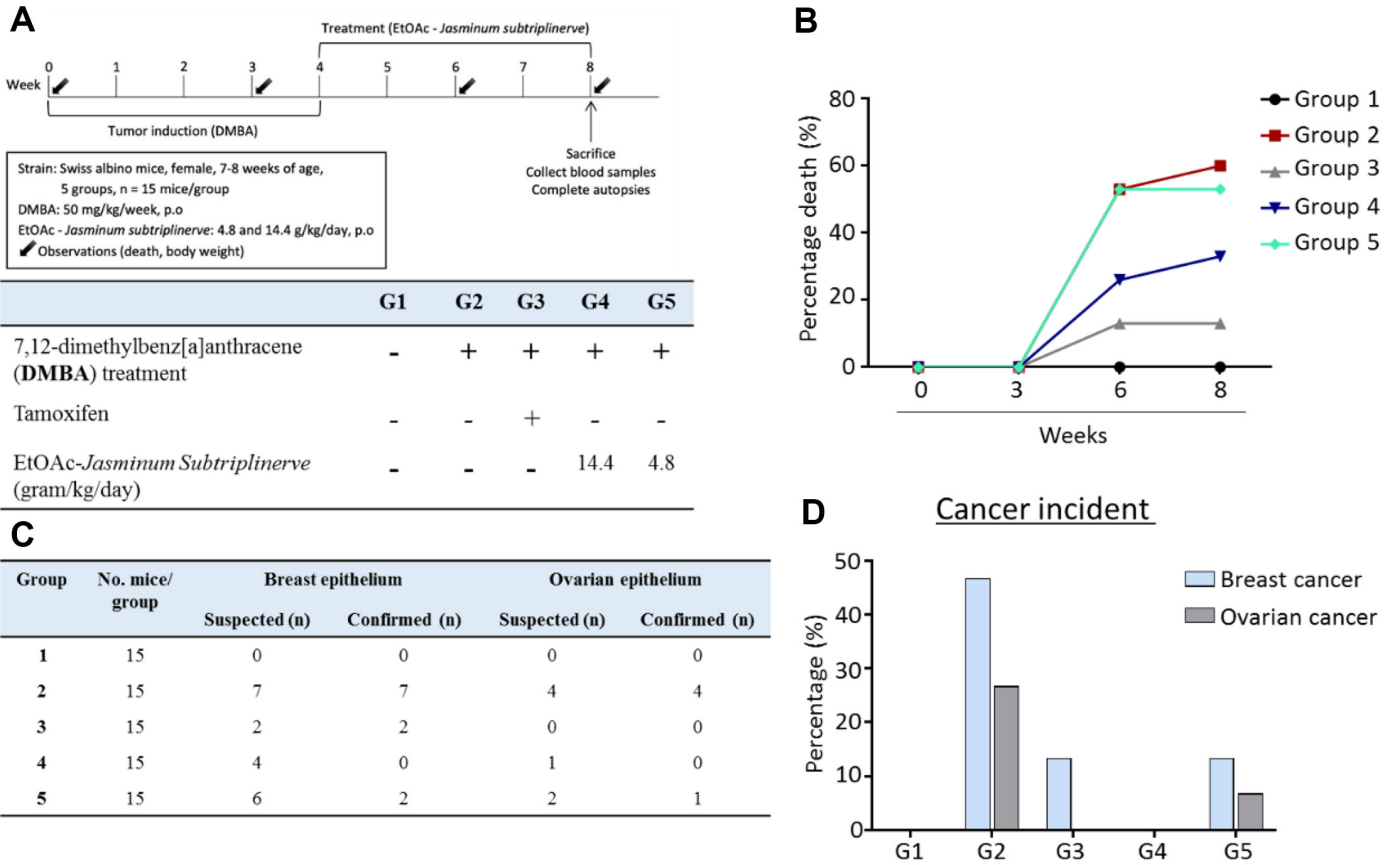
Effects of *J. subtriplinerve* fractionated extract (EtOAc) on clinical symptoms in a mouse model of DMBA-induced cancer. Clinical symptoms were observed during the experimental period. (**A**) Experimental scheme. (**B**) Survival rate. (**C**) Number of mice suspected to have tumors and confirmed via H&E analysis. (**D**) Cancer incidence. Data are presented as means ± standard deviations of 15 mice per group. Statistical significance was assessed via one-way ANOVA followed by Bonferroni’s post hoc test; *p < 0.05, **p < 0.01, vs. group 2.

DMBA was orally administered for 4 weeks before the samples were treated. Throughout the experiment, all mice were monitored daily for signs of tumors and mortality. As shown in Fig. 3, approximately 60% of mice in group 2 (DMBA administration only) died. Treatment with a dose of 4.8 mg/kg fractionated extract did not significantly reduce mortality: 53.3% of the mice died. However, a high dose of 14.4 mg/kg fractionated extract or tamoxifen strongly reduced mortality to 33% and 13%, respectively.

The numbers of mice that died with suspected tumors were recorded; tissue samples were collected for histological analysis to confirm the cause of death in each group. In group 2, all mice with suspected breast or ovarian tumors were subsequently confirmed to have cancer (46.67% breast cancer and 26.7% ovarian cancer)(Fig. 3). In Group 3 (tamoxifen treatment), two mice were confirmed to have breast cancer; none had ovarian cancer. Similarly, in group 5, two of six and one of two mice were suspected to have breast cancer and ovarian cancer, respectively. No cases of cancer were histologically detected after 4 weeks of treatment with high doses of the extract fraction in group 4, although four mice had previously been suspected to have breast tumors.

### Effects of *J. subtriplinerve* on DMBA-Induced Histopathological Changes in Mammary and Ovarian Tissues

**Fig. 4:**
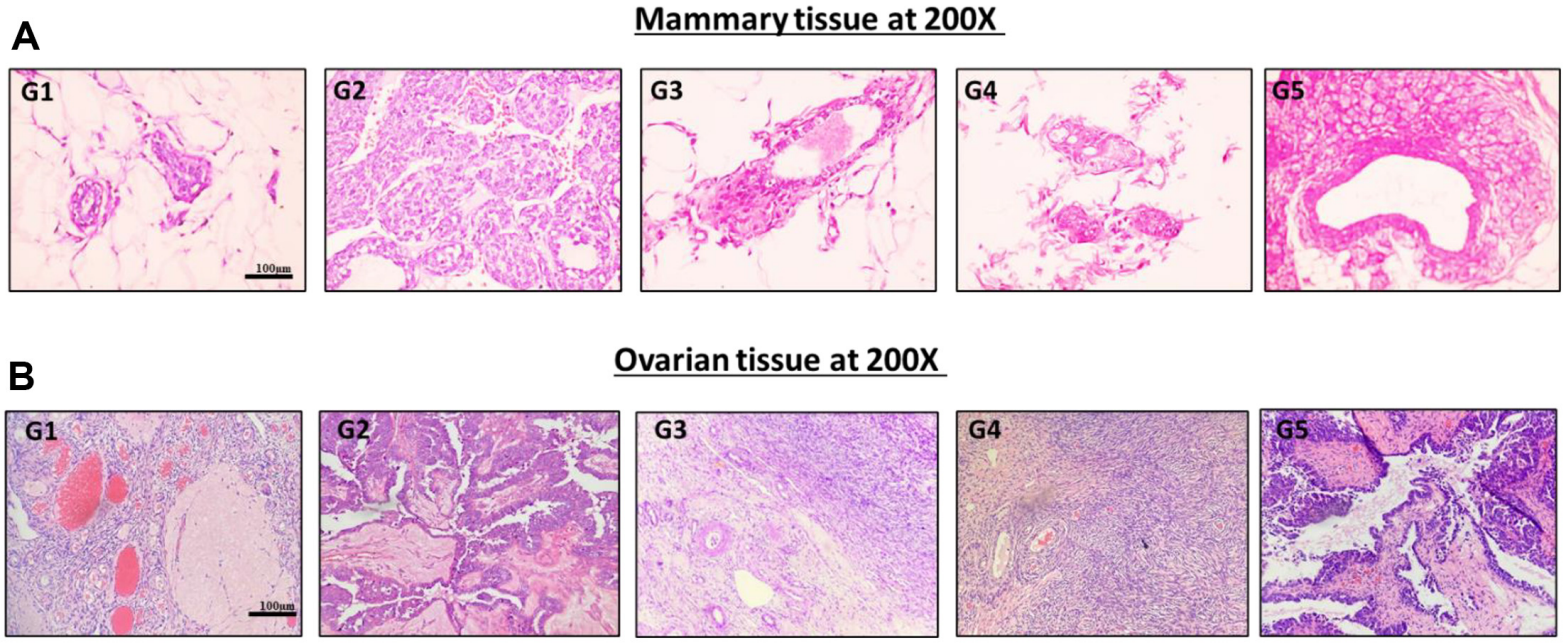
Changes in histological sections. Histological sections of mouse mammary tissues and ovarian tissues were viewed under 200 × magnification after H&E staining. (**A**) Groups 1 and 4 showed normal mammary gland structures, with small mammary ducts surrounded by a small amount of fibrous connective tissue. Group 2 showed various histopathological changes such as hyperplasia in some ducts, mild ductal proliferations, and focal epithelial hyperplasia with enlarged, hyperchromatic nuclei. Group 5 exhibited dysplastic mammary glands, an increase in channel number, and irregular cell division. (**B**) Normal ovarian structure in groups 1 (controls) and 4. Group 2 displayed diffuse tumors that disrupted the normal ovarian histological structure. Cells were large and had irregular nuclei, coarse chromatin, and a high nucleus-tocytoplasm ratio. They also showed a high rate of division.

Histological sections of mammary tissues from the control group showed a normal mammary gland structure, with small mammary ducts surrounded by a small amount of fibrous connective tissue (Fig. 4). In contrast, mice in the DMBA group exhibited various histopathological changes. Typical findings included hyperplasia in some ducts, mild ductal proliferations, and focal epithelial hyperplasia with hyperchromatic enlarged nuclei. Tissues in the extract treatment groups showed a normal structure, although group 5 displayed dysplastic mammary glands with increased duct number and irregular cell division. Ovarian tissues in controls and group 4 mice (treated with *J. subtriplinerve*) showed normal ovarian structure. In contrast, group 2 displayed diffuse tumors that disrupted the normal ovarian histological structure. The cells were large and had irregular nuclei, coarse chromatin, and a high nucleus-to-cytoplasm ratio. The rate of division was high.

### Lymphocytosis in Tissue

**Fig. 5:**
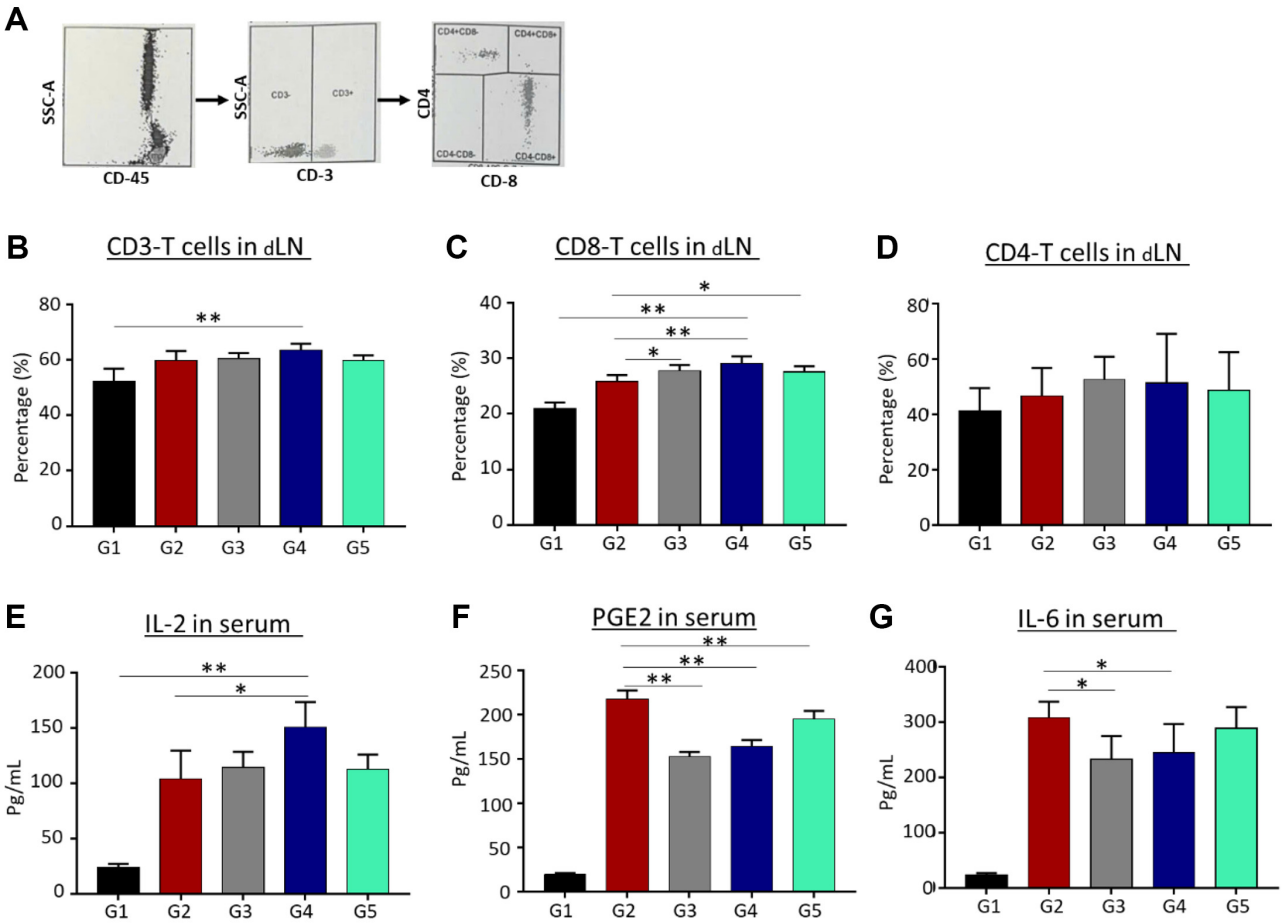
Effects of extracts on cytokines and lymphocytes. Treated mice were dissected 1 day after the last treatment. Blood and draining lymph nodes were collected for tests. (**A**) The gating of CD3, CD8, CD4 T cells by flow cytometer analysis, (**B**) percentage CD3 T cells, (**C**) percentage CD8 T cells and (**D**) percentage CD4 T cells were counted by CD3-CD4-CD8 test. The level of (**E**) IL-2 and (**F**) PGE2, (**G**) IL-6 expression in serum were measured by ELISA. Data are presented as means ± standard deviations of three mice per group. Statistical significance was assessed via one-way ANOVA followed by Bonferroni’s post hoc test; *p < 0.05, **p < 0.01, vs. group 2.

Flow cytometry of lymphocytes using the CD3-CD4-CD8 count test showed that compared with the vehicle group, DMBA treatment significantly increased the percentages of CD3 and CD8 T cells Fig. 5A and 5B to Fig. 5B and 5C, respectively no differences was observed in CD4 T cells compared to group 2, even hough it’s percentage was higher than group 1 (Fig. 5D). Furthermore, treatment with the fractionated extract in groups 3 and 4 resulted in higher percentages of CD8 T cells in the blood compared with the untreated group.

### Effects of Extracts on Th1 Cytokine Levels in Serum

IL-2 levels, determined using an ELISA kit, rapidly increased after DMBA treatment compared with the levels in controls (group 1). After 4 weeks of treatment with a high dose of the extract fraction (14.4 mg/kg; group 4), the levels increased even more noticeably. PGE2 and IL-6 levels in serum also increased in response to DMBA; tamoxifen and the higher dose of extract fraction significantly inhibited this increase.

### Dose Toxicity Effects

**Fig. 6:**
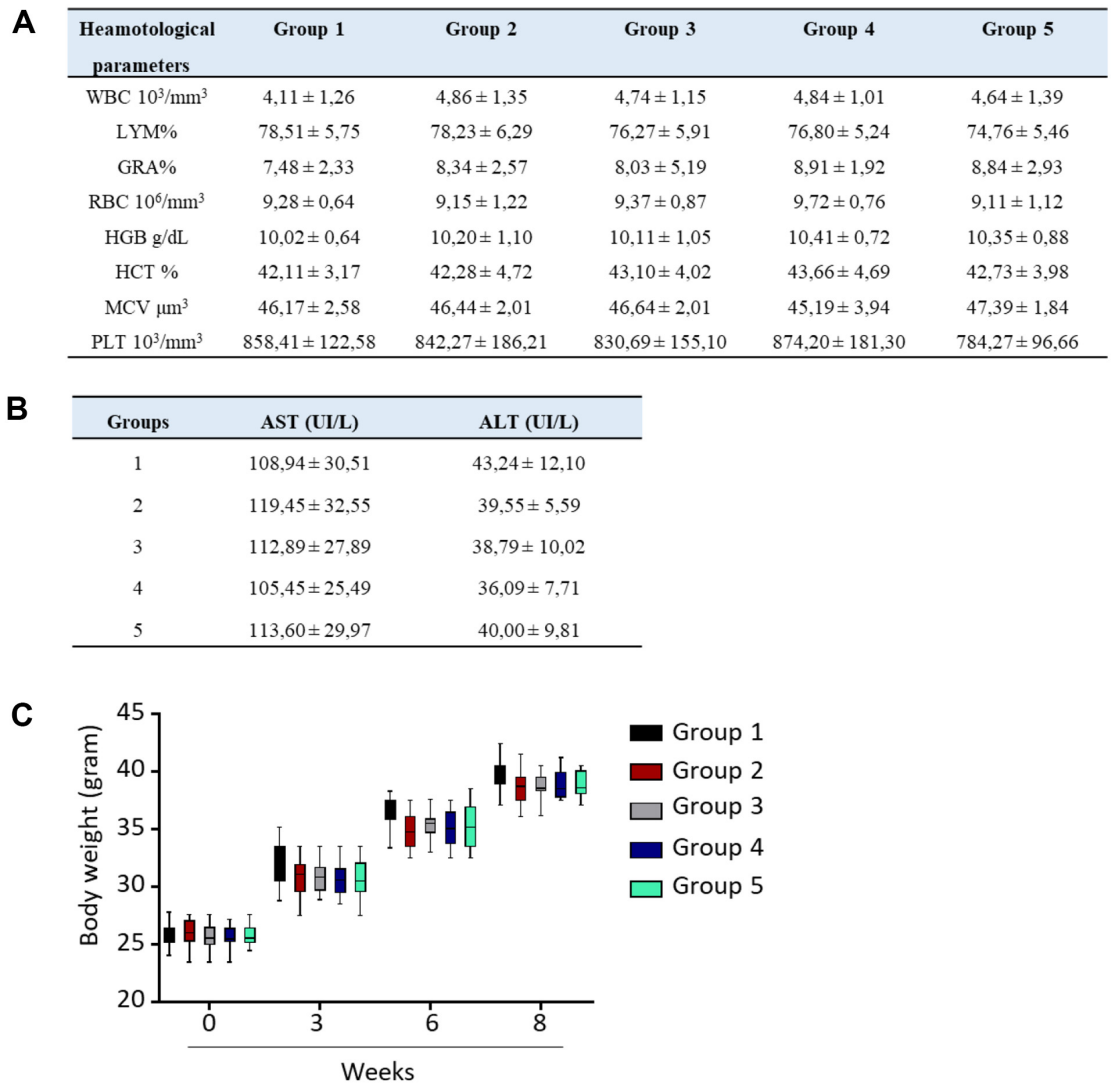
Dose toxicity effects. Female mice were administered *J. subtriplinerve* daily for 4 weeks and clinical observations were made. (**A**) Changes in hematological results. (**B**) Levels of liver marker enzymes. (**C**) Changes in body weight.

Treatment with different doses of the extract fraction had no effect on hematological parameters, and there were no changes in serum levels of ALT or AST compared with controls (Fig. 6). Additionally, no abnormal changes in body weight were noted.

### Identification of the Active Compound with Anticancer Activity against MCF-7

**Fig. 7:**
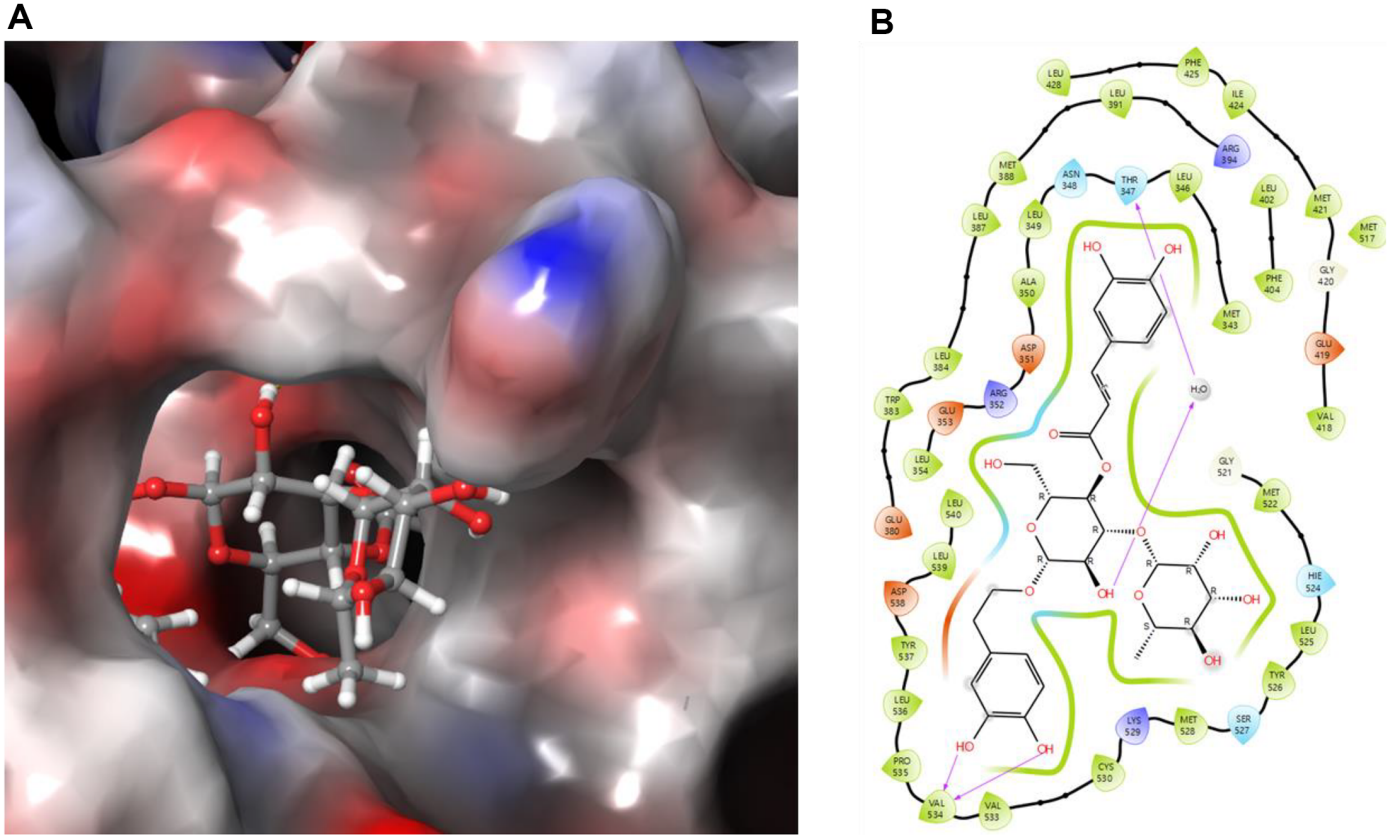
Molecular docking simulation of acteoside with 3ERT. (**A**) Three-dimensional binding interactions and (**B**) two-dimensional diagram of observed ligand–receptor interactions between acteoside and breast cancer protein (3ERT).

To investigate the underlying mechanisms of the anticancer effects of *J. subtriplinerve* extract, we performed molecular docking simulations using Maestro v. 13.4.134 (Schrödinger software, MMshare v. 6.0.134, released 2022-4, Platform Windows-x64) [19]. The crystal structure of the breast cancer target was downloaded from the Protein Data Bank (PDB ID: 3ERT). Then, the compound detected in the highest amount, acteoside, was identified by LC-QTOF-MS and TLC. Acteoside was docked into the active site of mechanistic target 3ERT; the results indicated that it bound to the active site of 3ERT with a docking score of −11.949 kcal/mol, which was significant compared to ellipticine. Notably, THR347 and VAL534 induced and stabilized the active conformation of 3ERT (Fig. 7). Based on its high binding affinity energies, hydrogen bond interactions, and ability to generate the ligand-binding pocket, acteoside from *J. subtriplinerve* shows inhibitory potential in breast cancer treatment.

## Discussion

The MTT assay is an uncomplicated method for evaluating the inhibitory potential of natural extracts or pure compounds with potential anticancer properties [20]. Thus, it was used to assess the degree of inhibition of *J. subtriplinerve* crude extracts and fractions (in EtOAc, *n*-hexane, and *n*-BuOH) against three cancer cell lines. All preparations significantly inhibited MCF-7 breast cancer cells with IC_50_ values lower than 50 μg/ml. Ellipticine, a well-known anticancer agent, was used as a reference for comparison [21].

The IC_50_ value of ellipticine is approximately 1 μg/ml, whereas the EtOAc fraction of *J. subtriplinerve* had a value of 13.70 ± 0.93 μg/ml. This difference highlights the need for further comprehensive chemical studies to isolate pure compounds that may possess enhanced anticancer properties. To elucidate the anticancer effects of the EtOAc fraction, we administered this fraction to mice, daily by oral gavage for 4 weeks. At high doses (14.4 mg/kg), the mortality rate and cancer incidence both were significantly reduced. T cells play a crucial role in cancer pathogenesis and are categorized into two main types: TH1 and TH2. In immune responses, CD4 cells (*i.e.*, helper T cells) stimulate B cells to produce antibodies and activate cytotoxic T cells (CD8 cells). CD8 cells then directly participate in tumor destruction; indeed, these cells are under consideration for use in tumor vaccines [22]. In the present study, mice with DMBA-induced cancer exhibited a significant increase in the percentage of CD3 T cells compared with controls. We also evaluated the percentages of CD4 and CD8 T cells, the latter of which showed a significant increase in the cancer groups. Group 4, treated with a high dose of the EtOAc extract, showed the highest percentage of CD8 cells; this was significantly higher than the percentage in the negative control group. CD8 T cells function by directly attacking virus-infected cells and cancer cells [23, 24]. Therefore, exploration of the impacts of extract fractions on the percentages of CD4 and CD8 T cells can help elucidate their effects on cell-mediated immune responses, as well as the underlying anticancer mechanism.

In addition to immune organs and competent immune cells, the immune system involves extensive cytokine activity. Cytokines are important substances secreted by antigen-activated immune cells. In particular, IL-2, TNF-α, and IL-6 play crucial roles in the immune response to inflammation as well as cancer [23].

IL-2 is secreted by activated Th lymphocytes and stimulates both CD4 and CD8 T cells; it also promotes the development and differentiation of B lymphocytes by enhancing natural killer (NK) cells and lymphokine-activated killer (LAK) cells. IL-2 is secreted when an antigen binds to the T cell receptor (TCR) and induces the expression of IL-2 receptors (IL-2R). The subsequent interaction between IL-2 and IL-2R stimulates the growth, differentiation, and survival of Tc cells. IL-2/S4B6 immune complexes exhibit high stimulatory activity toward NK cells and CD8 T cells; they could potentially replace conventional IL-2 in cancer immunotherapy. This hypothesis is supported by the proportional increase in IL-2 secretion as CD8 T cell percentage increases during tumor-targeting therapy. Moreover, preclinical and clinical studies have demonstrated that IL-2 can augment the development of immune cells, enhancing their abilities to eliminate cancer cells, particularly in renal cell carcinoma and malignant melanoma. With appropriate IL-2 therapy, the cure rates for these two types of cancer can reach 18% for malignant melanoma and 37% for renal cell carcinoma [25, 26]. The EtOAc extract led to the highest levels of IL-2 and CD8 T cells, highlighting its anticancer potential. The inflammatory environment and immune response are closely associated with cancer progression and recurrence. In various types of tumors, development and metastasis are characterized by the epithelial–mesenchymal transition (EMT), initiation of tumor formation, and angiogenesis, processes that are increasingly linked to intrinsic or extrinsic inflammation. Among known inflammatory mediators, PGE2 increases the invasiveness and progression of epithelial tumors by promoting their growth, helping cells to evade apoptosis, activating the transcription of tyrosine kinase growth factor receptors, and inducing angiogenesis. Furthermore, it plays a critical role in the tumor microenvironment by suppressing antitumor immunity and regulating tumor immune evasion, thus supporting tumor progression [27].

IL-6 is a major pro-inflammatory cytokine involved in the inflammatory response and cancer progression. It is secreted by various types of immune cells, including T cells, macrophages, and tumor cells [1-3]. Its overexpression has been reported in almost all types of tumors. Normally, high levels of IL-6 in the tumor microenvironment are thought to reflect the relationship between inflammation and cancer. In this study, DMBA induced an increase in IL-6 levels in mice, but with the higher dose of EtOAc fraction treatment, a significant decrease in IL-6 was found. This also suggests this fraction’s ability to reduce inflammation or prevent cancer in future studies.

The development of drugs or functional foods for cancer prevention or treatment requires assessing their effectiveness in terms of inhibiting tumor growth while ensuring user safety. Our results indicate that after 4 weeks of continuous treatment with extracts of *J. subtriplinerve* leaves, there were no significant differences in blood and biochemical parameters between the treated and control groups (*i.e.*, the treatment was safe).

*J. subtriplinerve* belongs to the Oleaceae family; thus far, no study has revealed any toxicities associated with its use. For example, a study of *J. sambac* L. extracts demonstrated no acute oral toxicity at a dose of 5,000 mg/kg [28]. A study of *Litsea elliptica* demonstrated no acute oral toxicity at doses ranging from 400 to 5,000 mg/kg; moreover, doses of 125, 250, and 500 mg/kg administered daily for 28 consecutive days did not lead to changes in body weight, food intake, or water consumption [29]. Additionally, a 50% ethanol extract of *J. subtriplinerve* did not induce any signs of toxicity in mice at a maximum dose of 20 g/kg. Finally, a study showed that acteoside, a key compound in *J. subtriplinerve*, did not cause toxicity when administered at sub-chronic doses [30]. These findings, together with our results, support the safety and efficacy of this medicinal plant.

## Conclusion

Cancer is a leading cause of death worldwide, with millions of new cases diagnosed each year. Accordingly, there is a critical need to discover safe, effective, and natural anticancer agents that ensure safe, affordable, and accessible treatment. By focusing on natural compounds, researchers aim to develop therapies that effectively combat cancer while minimizing adverse side effects; this approach will make long-term treatment more tolerable and sustainable for patients.

Breast cancer is the third most common cancer among women, after lung and bronchial cancers. Despite significant diagnostic and therapeutic advancements in both industrialized and developing nations, breast cancer remains a leading cause of mortality among women [31]. This disease significantly threatens health and places substantial burdens on patients and society. Breast cancer typically originates in the milk ducts or lobules of breast tissue. Multiple internal and external factors can contribute to its development, including hormones, immune system factors, genetic mutations, chemicals, and radiation [32].

*J. subtriplinerve* belongs to the jasmine family (Oleaceae) and is widely distributed, particularly in southern Asia. In traditional Vietnamese medicine, this plant is renowned for its therapeutic effects in terms of clearing heat, relieving rheumatism, promoting blood circulation, regulating menstruation, and reducing inflammation [9]. It is used to treat conditions such as amenorrhea, metritis, mastitis, rheumatism, and jaundice. It also supports liver function, increases bile secretion, improves digestion, stimulates appetite, and promotes restful sleep [8]. It has antibacterial and anti-inflammatory properties and is used to treat rheumatism, bone and joint pain, skin ailments, and snake bites. Its leaves are used in the management of irregular menstruation, high postpartum fever in women, lymphadenitis, metritis, mastitis, breast abscess, vaginal discharge, rheumatism with bone and joint pain, scabies, sores, impetigo, and various itchy skin conditions.

We comprehensively assessed the use of *J. subtriplinerve* as treatment for cancer and found that a 70% ethanol extract showed chemopreventive efficacy in a mouse model of breast cancer. Our results suggest that *J. subtriplinerve* extract can help to prevent the development of breast cancer without causing toxicity.

## Supplemental Materials
