# Supplementary material for: Efficacy of Jasminum subtriplinerve Extract against 7,12-Dimethylbenz[α]anthracene-Induced Cancer in Mice
Source: J Microbiol Biotechnol. 2024 Sep 20;34(11):2173–83. doi: 10.4014/jmb.2407.07045 (PMC11637824; doi:10.4014/jmb.2407.07045)

J. Microbiol. Biotechnol. 2024. 34(11): 2173–2183

https://doi.org/10.4014/jmb.2407.07045

Supplementary Figures

Efficacy of *Jasminum subtriplinerve* extract against 7,12-dimethylbenz[*a*]anthracene-induced cancer in mice

Phan Hong Minh [1, 2], Pham Thi Van Anh [2], Bui Thanh Tung [1], Ho My Dung [1], Tran Thi Thu Trang [6], Pham Thi Hong Nhung [1], Nguyen Thi Hang [3], Nguyen Thi Minh Nguyet [4], Nguyen Viet Phong [5], Le Ba Vinh5[*], and Mai Phuong Thanh [2]

[1] *University of Medicine and Pharmacy, Vietnam National University, Hanoi, Vietnam*

[2] *Department of Pharmacology, Hanoi Medical University, Hanoi, Vietnam*

[3] *National Institute of Medical Materials (NIMM), Hanoi, 11022, Vietnam*

[4] *Vinmec- VinUni Institute of Immunology, Vinmec Healthcare System, Hanoi, Vietnam*

[5] *Institute of Marine Biochemistry, Vietnam Academy of Science and Technology, Hanoi, Vietnam*

[6] *Hanoi University Pharmacy, Hanoi, Vietnam*

*Corresponding author.

Prof. Mai Phuong Thanh.

E-mail address: *maiphuongthanh@hmu.edu.vn* (M.P. Thanh).

Dr. Le Ba Vinh.

E-mail address: *lebavinh.imbc@gmail.com* (L.B. Vinh).

1. Cell viability on normal cells (RAW 264.7 cells)

The safety of compound on cell viability was determined by an MTT assay. The macrophage murine cells (RAW 264.7) were seeded into a 96-well plate at a density of $1x10^5$ cells/ well. Different concentrations of EtOAc fraction ranging from 6.25 µM to 100 µM, respectively were treated for cells for 6h. Subsequently, 10 µL of MTT stock (Sigma Aldrich) (5mg/ml) was added to each well for 2h. After removing the supernatant, the formazan crystals were dissolved by 100µl of DMSO at room temperature. The absorbance was measured by microreader at 570nm.

2. Toxicology dose

Female Swiss albino mice aged 6 weeks of age were divided into two groups. All mice in group 1 were treated with 200µL of saline solution, whereas mice in group 2 were administered with 200µL EtOAc fraction of *J. subtriplinerve* daily for 8 weeks. Mice were sacrificed 1 day after the last dose. Changes in body weight and survival rate were measured during experimental period. Blood samples were collected for assays hematological parameters analysis.

Supple Fig S1. Cytotoxicity on Raw 264.7 murine macrophage cells.

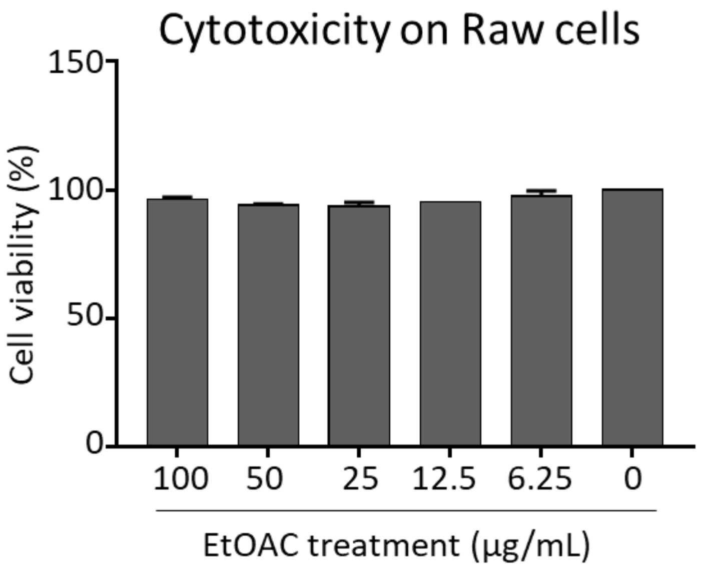

Supple Fig S2. Dose toxicity effects. Female mice were administered *J. subtriplinerve* daily for 8 weeks and clinical observations were made. (**A**) Changes in body weight. **(B)** Changes in hematological results. (**C**) Survival rate. (**D**) Changes in liver and kidney tissues.

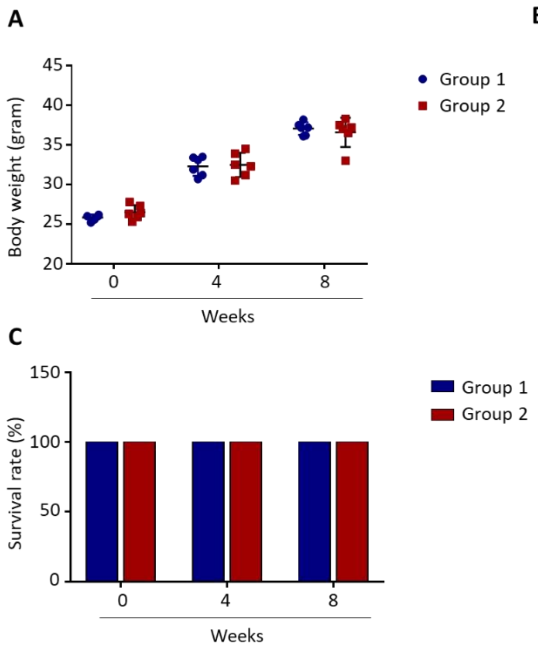

B

	RBC $10^6/mm^3$	MCV μm^3	HCT %	HGB g/dL	WBC $10^3/mm^3$	PLT $10^3/mm^3$
**Group 1**	9.69±0.35	45.83±0.93	42.66±1.14	10.31±0.27	4.16±0.07	846.23±10.04
**Group 2**	9.79±0.16	46.41±0.64	43.17±0.89	10.79±0.16	4.52±0.04	857.79±14.85

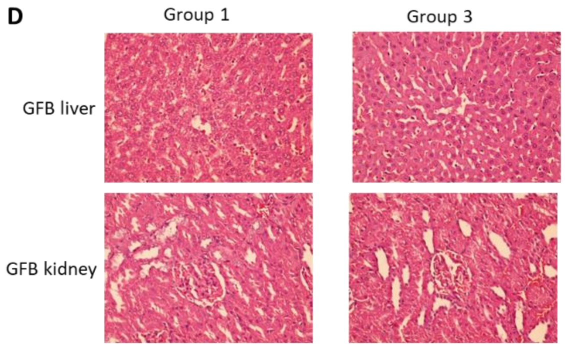

Supplement: Supplementary file 1 [file jmb-34-11-2173-supple.pdf]
